# Supplementary material for: First Isolation of Virulent Tenacibaculum maritimum Isolates from Diseased Orbicular Batfish (Platax orbicularis) Farmed in Tahiti Island
Source: Pathogens. 2022 Jan 21;11(2):131. doi: 10.3390/pathogens11020131 (PMC8877024; doi:10.3390/pathogens11020131)
Supplement: Supplementary file 1 [file pathogens-11-00131-s001.zip › Lopez et al 11-11-21 Supplementary Materials.pdf]

Figure S1. Phylogenetic relationships of 16S rDNA nucleotide sequences of the strains recovered in this study and the type strains of all *Tenacibaculum* species (See Table S2 for accession number). The phylogenetic tree was constructed with the MAFFT online tool using the neighbor-joining (NJ) method with the Jukes Cantor substitution model on all gap-free sites (1316pb) based on an alignment of 32 members of the genus *Tenacibaculum* performed with the L-INS-i method. Numbers at each branch indicated the percentage bootstrap values on 1,000 replicates. The 16S rDNA sequence of *Pseudotenacibaculum haliotis* (strain FDZSB0410) was used as an outgroup.

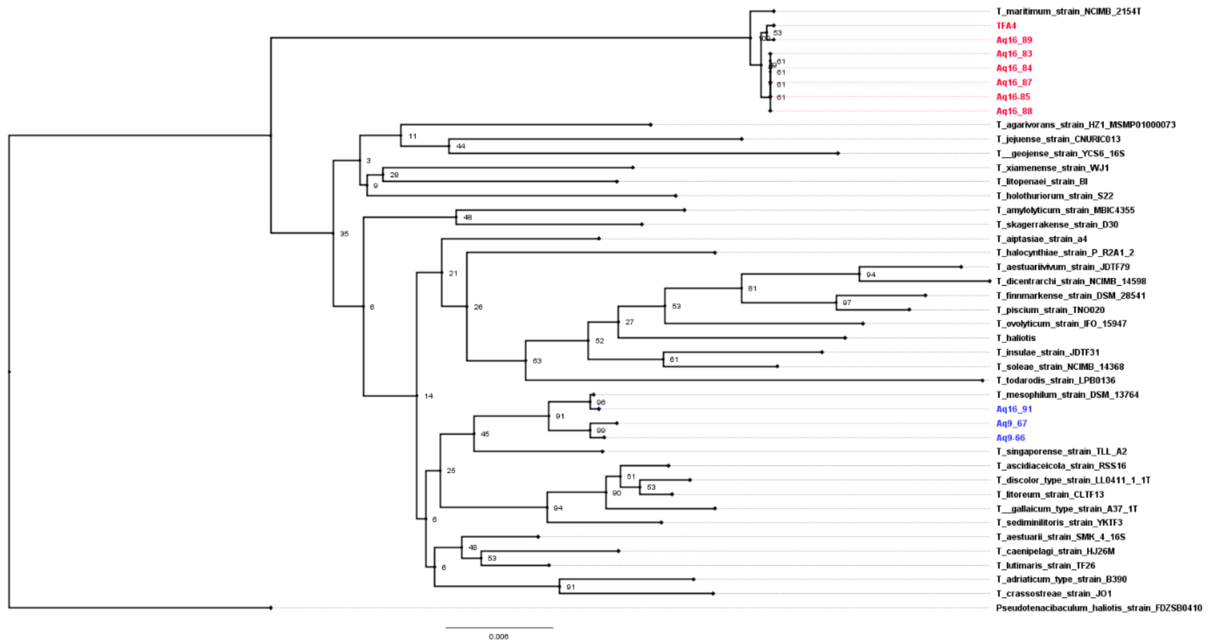

Table S1. List of PCR primers and probe used in this study.

Name	Target gene	Sequence (5′-3′)	Reference
27F	*16S rDNA*	AGAGTTTGATCMTGGCTCAG	Universal sequencing primer
1492R	*16S rDNA*	TACGGYTACCTTGTTACGACTT	Universal sequencing primer
785F	*16S rDNA*	GGATTAGATACCCTGGTA	Universal sequencing primer
805R	*16S rDNA*	GACTACCAGGGTATCTAATC	Universal sequencing primer
518R	*16S rDNA*	GTATTACCGCGGCTGCTGG	Universal sequencing primer
1100F	*16S rDNA*	YAACGAGCGCAACCC	Universal sequencing primer
MAR 4 fwd	*16S rDNA*	TGCCTTCTACAGAGGGATAGCC	Fringuelli et al. (2012) [35]
MAR rev	*16S rDNA*	CTATCGTTGCCATGGTAAGCCG	Fringuelli et al. (2012) [35]
MAR probe	*16S rDNA*	Texas Red -CACTTTGGAATGGCATCG-BHQ2	Fringuelli et al. (2012) [35]

Table S2. Accession number of the type strains used in Figure S1.

**Type strains**	**Genome accession number**
Tenacibaculum adriaticum B390^T	AM412314
Tenacibaculum aestuarii SMK-4^T	DQ314760
Tenacibaculum aestuariivivum JDTF-79^T	MF193601

Tenacibaculum agarivorans HZ1^{T}	MSMP01000073
Tenacibaculum aiptasiae a4^{T}	EF416572
Tenacibaculum amylolyticum MBIC4355^{T}	AB032505
Tenacibaculum ascidiaceicola RSS1-6^{T}	KT231981
Tenacibaculum caenipelagi HJ-26M^{T}	KC832834
Tenacibaculum crassostreae JO-1^{T}	EU428783
Tenacibaculum dicentrarchi 35/09^{T}	FN545354
Tenacibaculum discolor DSM 18842^{T}	RCCS01000002
Tenacibaculum finnmarkense DSM 28541^{T}	KT270385
Tenacibaculum gallaicum A37.1^{T}	AM746477
Tenacibaculum geojense YCS-6^{T}	HQ401023
Tenacibaculum halocynthiae P-R2A1-2^{T}	JX912707
Tenacibaculum haliotis RA3-2^{T}	KX450476
Tenacibaculum holothuriorum S2-2^{T}	LAPZ01000023
Tenacibaculum insulae JDTF-31^{T}	MF765760.1
Tenacibaculum jejuense KCTC 22618^{T}	LT899436
Tenacibaculum litopenaei B-I^{T}	DQ822567
Tenacibaculum litoreum CL-TF13^{T}	AY962294
Tenacibaculum lutimaris DSM 16505^{T}	RAQM01000002
Tenacibaculum maritimum NCIMB 2154^{T}	KT270382.1
Tenacibaculum mesophilum DSM 13764^{T}	jgi.1107970
Tenacibaculum ovolyticum IFO 15947^{T}	AB078058
Tenacibaculum piscium TNO020	GU124766
Tenacibaculum sediminilitoris YKTF-3^{T}	KU696540
Tenacibaculum singaporense TLL-A2^{T}	MG641897
Tenacibaculum skagerrakense D30T	AF469612
Tenacibaculum soleae LL04 12.1.7^{T}	AM746476
Tenacibaculum todarodis LPB0136^{T}	CP018155
Tenacibaculum xiamenense WJ-1^{T}	JX984443

Reference

35. Fringuelli, E.; Savage, P.D.; Gordon, A.; Baxter, E.J.; Rodger, H.D.; Graham, D.A. Development of a Quantitative Real-Time PCR for the Detection of *Tenacibaculum maritimum* and Its Application to Field Samples: Real-Time PCR for *Tenacibaculum maritimum*. *J Fish Dis* **2012**, *35*, 579–590, doi:10.1111/j.1365-2761.2012.01377.x.
